# Werner Syndrome Caused by Homozygous Frameshift Variant c.1578del in *WRN*

**DOI:** 10.15388/Amed.2024.31.2.9

**Published:** 2024-12-04

**Authors:** Jovita Patricija Druta, Gunda Petraitytė, Aušra Sasnauskienė, Eglė Preikšaitienė

**Affiliations:** 1 Faculty of Medicine, Vilnius University, Vilnius, Lithuania; 2 Department of Human and Medical Genetics, Institute of Biomedical Sciences, Faculty of Medicine, Vilnius University, Vilnius, Lithuania; 3 Department of Biochemistry and Molecular Biology, Institute of Biosciences, Life Sciences Centre, Vilnius University, Vilnius, Lithuania

**Keywords:** Progeroid syndromes, Werner syndrome, *WRN* gene, premature aging, Raktažodžiai: progerijos sindromai, Vernerio sindromas, *WRN* genas, ankstyvas senėjimas

## Abstract

**Background:**

Progerias are rare hereditary genetic disorders that cause the onset of aging to occur earlier than generally expected, which initiates the progression of many age-related diseases. Syndromes assigned to this group are usually a compound disturbance of multiple systems. Werner syndrome is among a few well described premature aging disorders associated with a higher likelihood of malignancies.

**Clinical case:**

We present a 45-year-old man with a history of painful muscle spasms, general muscle pain and weakness. There was a progression of contractures of the plantar tendons, as well as the atrophy of the subcutaneous adipose tissue of the extremities. The patient was initially diagnosed with secondary small fiber sensory polyneuropathy and myotonia, but further genetic testing revealed the homozygous pathogenic variant c.1578del in the *WRN* gene associated with Werner syndrome.

**Conclusions:**

The c.1578del variant, previously not described in literature in a homozygous state, causes Werner syndrome and is associated with the pronounced hallmarks of early senescence in the proband’s fibroblasts. Molecular diagnosis brings better treatment of manifestations and monitoring options for the patients, helping to establish more sufficient and secure patient care.

## Abstract (it)

**Įvadas:**

Progerija yra reta paveldima genetinė liga, dėl kurios senėjimas prasideda anksčiau, nei paprastai tikimasi, ir dėl to progresuoja daugelis su amžiumi susijusių ligų. Šiai grupei priskiriami sindromai paprastai yra sudėtinis kelių sistemų sutrikimas. Vernerio sindromas yra vienas iš keleto gerai aprašytų ankstyvo senėjimo sutrikimų, susijusių su didesne piktybinių navikų tikimybe.

**Klinikinis atvejis:**

45 metų vyrui, kuriam pasireiškė skausmingi raumenų spazmai, bendras raumenų skausmas ir silpnumas. Progresavo padų sausgyslių kontraktūros, taip pat galūnių poodinio riebalinio audinio atrofija. Iš pradžių pacientui buvo diagnozuota antrinė smulkiųjų skaidulų sensorinė polineuropatija ir miotonija, tačiau tolesni genetiniai tyrimai nustatė homozigotinį patogeninį *WRN* geno c.1578del variantą, susijusį su Vernerio sindromu.

**Išvados:**

Variantas c.1578del, anksčiau literatūroje neaprašytas homozigotinėje būsenoje, sukelia Vernerio sindromą ir yra susijęs su ryškiais ankstyvo senėjimo požymiais probando fibroblastuose. Molekulinė diagnostika suteikia geresnių pacientų simptomų gydymo ir stebėsenos galimybių, padedančių nustatyti pakankamą ir saugesnę pacientų priežiūrą.

## Introduction

Progeroid syndromes are a group of rare, hereditary, severe disorders that are often described as premature aging. These conditions often have an early onset and lead to a shortened lifespan. Examples of these disorders are Hutchinson–Gilford progeria syndrome (MIM 176670), Fanconi anemia (MIM 227650, 617243, 616435, 614083, 227646, 600901, 617247, 614082, 227645, 603467, 605724, 617244, 609053, 613951, 615272, 610832, 617784, 227650, 617883, 613390, 609054, 300514), Rothmund–Thomson syndrome (MIM 268400, 615789, 618625, 620819), Cockayne’s syndrome (MIM 216400, 133540), xeroderma pigmentosum (MIM 278730, 278760, 278780, 278700, 278720, 278740. 278750, 610651), and Werner syndrome (MIM 277700) [1]. Hutchinson–Gilford progeria syndrome, as one of the most studied conditions in early-onset progeroid syndromes, has a birth prevalence of around 1 to 4 in 8 million, while Werner syndrome, also known as adult progeria, has an incidence of around 1 in 1 million births, though many cases are undiagnosed [2, 3]. Clinical features commonly associated with these disorders are early graying and loss of hair, cataracts, fragile skin, mandibular hypoplasia, lipodystrophy, contractures, decreased joint mobility, skeletal abnormalities, and growth retardation appearing in different stages of life. Accelerated aging also comes with a more rapid and early progression of age-related health conditions such as atherosclerosis, sarcopenia, osteoporosis, diabetes mellitus, and metabolic impairment. Syndromes can also appear in atypical forms that make them even more complicated for a clinician to suspect and take diagnostic measures.

Werner syndrome is among a few well described premature aging disorders manifesting in late adolescence or early adulthood. Individuals diagnosed with the condition often tend to have specific facial features, such as beaked nose, micrognathia, and a high-pitched voice. As the disease starts progressing in later years of life, cognitive development and abilities remain unaffected. Werner syndrome is also among the syndromes associated with a higher likelihood of malignancies [4].

Werner syndrome is associated with biallelic pathogenic variants in the *WRN* gene (also known as *RECQL2*), which is located in chromosome 8p12. The gene encodes a WRN protein that is a RecQ DNA helicase, exonuclease, and ATPase. The RecQ helicase family consists of proteins in charge of DNA metabolism, also commonly referred to as DNA helicases. To date, the ones most prominently mentioned in the literature, other than WRN, are RECQL1, BLM, RECQL4 and RECQL5. Members differ in structural components as well as functional features, yet all share an important role in DNA replication, transcription, recombination, and the repair process [5-7]. Genetic errors in the genes coding those enzymes cause the manifestation of molecular features like telomere shortening, stem cell exhaustion, and genomic instability, which are responsible for aging and aging-related health disturbances.

Dysfunction of the telomeres is considered to be among the key factors in the occurrence and progression of the conditions usually referred to as age-related diseases. The phenomenon is associated with a possible suppressive influence on oncogenesis since it is accountable for shorter lifespan of the cell constant readjustment of DNA sequence conditions ongoing genomic modifications [8]. Alteration that has been proven to hold a strong link to promoting oncogenic activity as well as manifestation of accelerated aging syndromes are errors in DNA repair system which play a role in genomic instability [9, 10]. Perturbations in vitality and longevity are also partially caused by stem cell exhaustion. Over time cell renewal ability is lost due to misconduct of stem cell reproduction and proliferation, leading to cell senescence and overall aging [11].

We report a Lithuanian patient with a homozygous pathogenic variant in the *WRN* gene that led to Werner syndrome, which was detected in the patient’s late adulthood.

## Materials and methods

### Clinical Evaluation

The study was approved by the Vilnius Regional Biomedical Research Ethics Committee of Lithuania (protocol code No. 2021/9-1373-849, date of approval 21 September 2021).

A 45-year-old man presented with a history of painful muscle spasms, general muscle pain, and weakness. Spontaneous muscle spasms started at the age of 10 years. An episode of diplopia in childhood was noted. At the age of 18 years, the proband was consulted by a neurologist, but no medical records are available. The proband reported a progression of symptoms through the years, including sensitivity of his feet and pain that caused difficulty walking. He experienced contractures of the plantar tendons and atrophy of the subcutaneous adipose tissue of the extremities. That same year arthrodesis of the right foot was performed. Due to necrosis of his third and fourth toes caused by a postoperative infection, amputation was conducted. At the age of 45 years, the proband was admitted to the Department of Neurology. Neurological examination showed percussion provoked myotonia of his palms and forearms and plantar hyperesthesia and allodynia. Several EMGs showed no signs of myopathy or myotonia. Blood analysis showed mild elevation of liver enzymes and lactate. Creatinine kinase was at a normal range. The patient was initially diagnosed with secondary small fiber sensory polyneuropathy and myotonia. The proband was previously diagnosed with hypopituitarism, hyperprolactinemia, hypogonadism, gout, and hypertension. At the age of 45 years, the proband was diagnosed with nontoxic multinodular goiter and hypothyroidism. His hair began turning gray at the age of 30. Over the preceding few years, he had developed a high-pitched voice.

On admission at age of 45 years, the proband’s height was 178 cm and his weight was 78 kg. Physical examination revealed micrognathia, a beaked nose, scleroderma-like skin on his face and extremities, slender limbs with atrophy of the subcutaneous tissue, abdominal obesity, deformation of his feet, contractures of his toes, and amputation of the third and fourth toes of his right foot.

### DNA Extraction

Genomic DNA (gDNA) was isolated from peripheral blood leukocytes of the proband using phenol–chloroform–isoamyl alcohol extraction method [12].

### Next-Generation Sequencing

Exome sequencing was performed on proband’s genomic DNA using HumanCore Exome Kit (Twist Bioscience), as described previously [6]. Genes associated with progeroid syndromes and neuromuscular disorders have been analyzed. Variants were classified following the guidelines of the American College of Medical Genetics and Genomics [13]. Only variants that passed quality and coverage filters and showed >99.9% detection reliability were analyzed.

### Cell senescence assay

Fibroblast cultures from the proband and two healthy controls (male and female) were used for a senescence assay. The cells were cultured in AmnioMAX C-100 medium (Gibco) supplemented with 13% specific medium supplement C-100 (Gibco) and 0.38% amphotericin B. Early cell senescence was detected using a commercial senescence cell histochemical staining kit (Sigma Aldrich) according to the manufacturer’s recommendations. After cell staining, at least ten images were captured at random sites of the cell culture by the EVOS FL auto cell imaging system (Thermo Fisher Scientific), using a 20× objective, followed by the quantification of teal-colored cells expressed as a percentage of the total amount of the cells.

## Results

Whole exome sequencing of proband’s DNA sample revealed a pathogenic homozygous variant of the *WRN* gene NM_000553.6:c.1578del, NP_000544.2: p.(Leu528CysfsTer29), rs780555196. This variant in compound heterozygous state with other pathogenic *WRN* variant has previously been described in individual showing signs of Werner syndrome [14]. *WRN* variant c.1578del is recorded in ClinVar as pathogenic / likely pathogenic [15]. The allele frequency in gnomAD v4.0.0 dataset exomes and genomes is 0.000023, homozygous allele count is 0. In our local database, *WRN* heterozygous variant c.1578del has been detected in 2 out of 1000 individuals.

**Figure 1:**
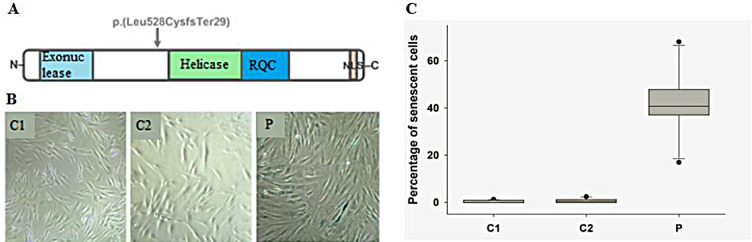
A. Domain structure of WRN and pathogenic variant p.(Leu528CysfsTer29) detected in the proband. B. Photographs of fibroblast cultures after incubation with X-gal substrate. C1, C2 are the cells of the control subjects and P are the cells of the proband. C. Percentage of cells in control individuals (C1, C2) and proband (P) with β-galactosidase activity characteristic of senescent cells.

Senescent cells are metabolically active and are identified by β-galactosidase activity during histochemical staining. We used this method to evaluate senescent cells. The assay showed that 56.72% of the proband’s fibroblasts were early senescent cells while only 0.43% of the control cells of the healthy female and 0.53% of the healthy male were positive for senescence-associated β-galactosidase activity (Figure 1). Owing to the progeroid phenotype exhibited by the proband and its consequential impact on cellular senescence and viability, a singular biological replicate was performed.

## Discussion

The proband’s fibroblasts analyzed in this study exhibited pronounced hallmarks of early senescence, which is consistent with findings reported in the literature. Cheung et al. [16] substantiated the pivotal role of telomerases in the senescence progression of fibroblasts derived from Werner syndrome patients harboring specific protein loss-of-function variants (e.g., R368X, Q748X), which exhibited pronounced early cellular senescence as indicated by β-galactosidase activity. Intriguingly, the same research team rectified one of the DNA variants (causing R368X) in induced pluripotent stem cells (iPSCs) obtained from Werner syndrome patients using CRISPR-Cas9 technology. Subsequent evaluation utilizing a cytochemical assay revealed a mitigated senescence phenotype [17].

To this day, there are approximately 278 pathogenic and likely-pathogenic variants discovered that have been associated with Werner syndrome. Most are protein-truncating variants, but missense variants have also been described. A few of the most frequent variants are c.1105C>T, c.3139-1G>C and c.2089-3024A>G. The most commonly diagnosed pathogenic variant worldwide is c.1105C>T, with Japan leading in number of cases detected. A change that is caused by this genetic alteration is a switch from arginine to a termination codon, which leads to an exonuclease 9 functional mismatch. The *WRN* variant c.3139-1G>C has more often been reported in a homozygous state and causes the loss of exon 26 [18]. The variant c.2089-3024A>G, with most reports coming from the population in Sardinia, exhibits the insertion of a new exon. According to many observations, loss of function is a known mechanism of disease. In the case presented, variant c.1578del was discovered previously in a heterozygous state [19] and is known to cause a premature stop signal that causes the formation of a defected protein. The parents of the affected individual resided in the same geographic area, i.e. North Zemaitija, but they were unavailable for further testing to support the hypothesis of a common shared ancestral allele.

Clinically, reports describing Werner syndrome have this list of pathologies mentioned: lipid metabolism disfunction, fertility issues, and dermatologic, ophthalmologic, endocrine, and orthopedic disorders. There is no significant difference in symptoms or laboratory or instrumental results when the most recent reports of the variants mentioned are compared. Anamnesis includes pain irradiating to different areas of the body, ulcers of the lower extremity, skin atrophy, lack of a pubertal growth spurt in the proband’s adolescent years, alopecia, hyperkeratosis, graying of hair and hoarseness appearing in the early or late twenties, bilateral cataracts, and hypogonadism. Laboratory results reveal lipid imbalance and altered glucose tolerance or diabetes mellitus; elevation of liver enzyme levels also is common. In terms of appearance, affected individuals share common facial structure features of petite head, beak shaped nose, hypoplastic mandibula, and retrognathia. Likewise, short height, thin limbs, and fat distribution most prominent in the abdominal area are usually observed. Instrumental investigations detected nonalcoholic fatty liver disease, low bone density, and calcifications located in the Achilles tendons [20-23]. Our proband also exhibits similar complaints and anamnesis with a more prominent set of endocrine pathologies: hypopituitarism, hyperprolactinemia, hypogonadism, hypothyroidism and gout. Additionally, Werner syndrome is associated with a higher probability and possible predisposition for the development of neoplastic processes, as published clinical reports include individuals with melanomas, meningiomas, soft-tissue sarcomas, and leukemia [24].

Clinicians should stay alert with regard to patients’ metabolic health and condition of skin and bones. Since most Werner patients are diagnosed with osteoporosis by the age of 40, it should be followed by a treatment strategy according to recommendations presented in the guidelines for treatment of osteoporosis, because there is no evidence for a different treatment plan for this specific group [25]. Skin atrophy and microcirculation disturbances lead to skin lesions, which are a good environment for infection to spread, and therefore careful inspection of extremities and adequate wound care is advised [26]. Clinical evidence shows that a common metabolic health issue in Werner syndrome patients, dyslipidemia, can be effectively controlled by the prescription of a strong statin [27]. Unfortunately, in many cases no prophylaxis or preventative measures can be taken, as the changes in one’s body are a result of early onset of aging, yet early diagnosis and appropriate treatment methods greatly benefit the quality of an individual’s life.

## Conclusion

The c.1578del variant in the *WRN* gene, previously not described in literature in a homozygous state, causes Werner syndrome and is associated with pronounced hallmarks of early senescence in the proband’s fibroblasts. Continuous updates and detection of new variants shed light on etiological, clinical, and diagnostic aspects, as well as treatment options for the patient, helping to establish a more sufficient and secure patient care system.

## References

[1] Talukder P, Saha A, Roy S, Ghosh G, Dutta Roy D, Barua S (2023). Progeria-a Rare Genetic Condition with Accelerated Ageing Process. Appl Biochem Biotechnol.

[2] Coppedè F (2021). Mutations Involved in Premature-Ageing Syndromes. Appl Clin Genet.

[3] Schnabel F, Kornak U, Wollnik B (2021). Premature aging disorders: A clinical and genetic compendium. Clin Genet.

[4] Hennekam RCM (2020). Pathophysiology of premature aging characteristics in Mendelian progeroid disorders. Eur J Med Genet.

[5] Gudmundsrud R, Skjånes TH, Gilmour BC, Caponio D, Lautrup S, Fang EF (2021). Crosstalk among DNA Damage, Mitochondrial Dysfunction, Impaired Mitophagy, Stem Cell Attrition, and Senescence in the Accelerated Ageing Disorder Werner Syndrome. Cytogenet Genome Res.

[6] Newman JA, Gileadi O (2020). RecQ helicases in DNA repair and cancer targets. Essays Biochem.

[7] Kategaya L, Perumal SK, Hager JH, Belmont LD (2019). Werner Syndrome Helicase Is Required for the Survival of Cancer Cells with Microsatellite Instability. iScience.

[8] Rossiello F, Jurk D, Passos JF, d’Adda di Fagagna F (2022). Telomere dysfunction in ageing and age-related diseases. Nat Cell Biol.

[9] López-Otín C, Blasco MA, Partridge L, Serrano M, Kroemer G (2023). Hallmarks of aging: An expanding universe. Cell.

[10] López-Otín C, Pietrocola F, Roiz-Valle D, Galluzzi L, Kroemer G (2023). Meta-hallmarks of aging and cancer. Cell Metab.

[11] Wang MJ, Chen J, Chen F (2019). Rejuvenating Strategies of Tissue-specific Stem Cells for Healthy Aging. Aging Dis.

[12] Javadi A, Shamaei M, Mohammadi Ziazi L (2014). Qualification study of two genomic DNA extraction methods in different clinical samples. Tanaffos.

[13] Grigaitė J, Šiaurytė K, Audronytė E (2021). Novel In-Frame Deletion in *HTRA1* Gene, Responsible for Stroke at a Young Age and Dementia-A Case Study. Genes (Basel).

[14] Richards S, Aziz N, Bale S (2015). Standards and guidelines for the interpretation of sequence variants: a joint consensus recommendation of the American College of Medical Genetics and Genomics and the Association for Molecular Pathology. Genet Med.

[15] Yokote K, Chanprasert S, Lee L (2017). WRN Mutation Update: Mutation Spectrum, Patient Registries, and Translational Prospects. Hum Mutat.

[16] Landrum MJ, Lee JM, Riley GR (2014). ClinVar: public archive of relationships among sequence variation and human phenotype. Nucleic Acids Res.

[17] Cheung HH, Liu X, Canterel-Thouennon L, Li L, Edmonson C, Rennert OM (2014). Telomerase protects Werner syndrome lineage-specific stem cells from premature aging. Stem Cell Reports.

[18] Tu J, Wan C, Zhang F (2020). Genetic correction of Werner syndrome gene reveals impaired pro-angiogenic function and HGF insufficiency in mesenchymal stem cells. Aging Cell.

[19] Matsumoto N, Ohta Y, Deguchi K (2019). Characteristic Clinical Features of Werner Syndrome with a Novel Compound Heterozygous WRN Mutation c1720+1G>A Plus c.3139-1G>C. Intern Med.

[20] Tsujimoto Y, Bando H (2023). Flame-like Calcifications in Werner Syndrome. JCEM Case Rep.

[21] Kinoshita D, Kato H, Kaneko H (2023). Case of Werner syndrome with significant improvement of refractory skin ulcer despite fibroblast cellular senescence. Geriatr Gerontol Int.

[22] Li H, Yang M, Shen H, Wang S, Cai H (2021). Severe metabolic disorders coexisting with Werner syndrome: a case report. Endocr J.

[23] Yamamoto R, Akasaki K, Horita M (2020). Evaluation of glucose tolerance and effect of dietary management on increased visceral fat in a patient with Werner syndrome. Endocr J.

[24] Lauper JM, Krause A, Vaughan TL, Monnat RJ (2013). Spectrum and risk of neoplasia in Werner syndrome: a systematic review. PLoS One.

[25] Mori S, Takemoto M, Kubota Y (2021). Management guideline for Werner syndrome 2020 4. Osteoporosis associated with Werner syndrome. Geriatr Gerontol Int.

[26] Taniguchi T, Takemoto M, Kubota Y (2021). Management guideline for Werner syndrome 2020 5. Infection associated with Werner syndrome. Geriatr Gerontol Int.

[27] Tsukamoto K, Takemoto M, Kubota Y (2021). Management guideline for Werner syndrome 2020 1 Dyslipidemia and fatty liver associated with Werner syndrome. Geriatr Gerontol Int.

